# Implementation of evidence-based personalized nursing care for Two patients With vasodepressor syncope: a case report

**DOI:** 10.3389/fcvm.2026.1780380

**Published:** 2026-07-13

**Authors:** Yunfeng Bai, Tianchao Chen, Yang Li, Jing Fu, Nan Wu, Xinjuan Wu

**Affiliations:** 1 Department of Nursing, Peking Union Medical College Hospital, Chinese Academy of Medical Science and Peking Union Medical College, Beijing, China; 2 Department of Cardiology, Peking Union Medical College Hospital, Chinese Academy of Medical Science and Peking Union Medical College, Beijing, China

**Keywords:** case report, continuity of care, EQ-5D-5L, personalized nursing care, reflex syncope, vasodepressor syncope

## Abstract

**Background:**

Vasodepressor syncope is a common subtype of reflex syncope. Although lifestyle modification, trigger avoidance, physical counterpressure manoeuvres, and orthostatic training are recommended, post-discharge nursing pathways that translate this evidence into individualized home-based care remain insufficiently described.

**Case presentation:**

We report two patients with tilt-test-confirmed vasodepressor syncope. Patient A was a 58-year-old man with recurrent brief syncopal episodes, fall-related injury, and insufficient prodrome recognition and self-monitoring. Patient B was a 74-year-old woman with recurrent dizziness and syncope-like episodes, blood pressure variability, cerebrovascular comorbidity, and higher anxiety related to unpredictable episodes.

**Intervention:**

An evidence-based personalized nursing care pathway was developed using guideline recommendations, syncope-related clinical studies, and individualized patient assessment. The intervention included lifestyle modification, fluid and salt intake guidance, physical counterpressure manoeuvre training, orthostatic/tilt training, blood pressure and heart-rate self-monitoring, trigger management, family involvement, and psychological support. Follow-up was performed by telephone and outpatient review over 3 weeks.

**Outcomes:**

No syncope recurrence, fall event, or other major discomfort occurred during the 3-week follow-up. Patient A showed improvement in EQ-5D-5L index from 0.720 to 0.906 and EQ VAS from 58 to 75; GAD-7 decreased from 8 to 5 and FES-I from 48 to 32. Patient B showed improvement in EQ-5D-5L index from 0.467 to 0.651 and EQ VAS from 48 to 66; GAD-7 decreased from 15 to 10 and FES-I from 44 to 36.

**Conclusion:**

A structured personalized nursing care pathway may support self-management, fall-prevention behaviours, and patient-reported outcomes in vasodepressor syncope. As this report involves only two cases and a short follow-up, the absence of recurrence should be interpreted as an early observation requiring prospective verification.

## 1 Introduction

Syncope is a transient loss of consciousness caused by transient global cerebral hypoperfusion and is characterized by rapid onset, short duration, and spontaneous complete recovery (1–3). Reflex syncope, including the vasodepressor phenotype, is among the most frequent forms encountered in clinical practice (1, 2, 4–6). In vasodepressor syncope, hypotension caused by inappropriate vasodilation and impaired autonomic compensation predominates, while marked cardio inhibition or prolonged bradycardia may be absent (1–3).

Guidelines and contemporary reviews consistently support education, reassurance, avoidance of triggers, adequate hydration and salt intake, physical counterpressure manoeuvres, and selected orthostatic training as first-line non-pharmacological management for reflex syncope (2, 4, 6–9). Evidence is also available for structured syncope services and nurse-led outpatient follow-up; a nurse-led syncope clinic has been associated with lower mortality and reduced re-hospitalization after syncope admission in a single-center retrospective cohort (10). However, several aspects remain insufficiently explored from a nursing perspective: how guideline-based recommendations should be translated into a practical discharge-to-home pathway, how interventions should be individualized according to triggers, lifestyle, family support, anxiety, and fear of falling, and how short-term patient-reported outcomes should be monitored after discharge.

The present case report was reorganized according to the CARE reporting framework for case reports (11). It describes the construction and clinical application of an evidence-based personalized nursing care pathway in two patients with vasodepressor syncope and illustrates how patient-specific needs can be linked to follow-up nursing interventions and measurable outcomes.

## 2 Methods and theoretical basis for personalized nursing care

### 2.1 Reporting framework

This report follows the CARE case-report structure, including patient information, clinical findings, timeline, diagnostic assessment, intervention, follow-up outcomes, discussion, and patient-centered interpretation (11).

### 2.2 Evidence search and synthesis

A literature search was conducted in PubMed, Cochrane Library, Embase, CINAHL, Wanfang Data, and CNKI for publications from January 1, 2015 to July 1, 2025. Search terms included syncope, vasovagal syncope, vasodepressor syncope, reflex syncope, nursing care, continuity of care, discharge planning, follow-up care, self-management, guideline, randomized controlled trial, systematic review, and evidence-based nursing. Classic guidelines and landmark trials published before 2015 were retained when they represented foundational evidence, including the PC-Trial and major guideline statements (2, 3, 6, 8).

The care pathway was built on three linked components: (1) best available evidence, including syncope guidelines, physical counterpressure manoeuvre trials, orthostatic training studies, and outpatient follow-up studies; (2) clinical expertise from cardiovascular nursing and evidence-based nursing; and (3) patient-specific needs, including triggers, comorbidities, self-management capacity, psychological burden, and family support. This clarified the terminology used throughout the manuscript: the overall programme is described as evidence-based personalized nursing care, while the actionable elements are described as nursing interventions.

### 2.3 Outcome measures

Outcomes were assessed at baseline and at the 3-week follow-up. Clinical outcomes included syncope recurrence, fall events, presyncope symptoms, and adverse events. Patient-reported outcomes included EQ-5D-5L index and EQ VAS for health-related quality of life, the Generalized Anxiety Disorder-7 (GAD-7) for anxiety symptoms, and the Falls Efficacy Scale-International (FES-I) for fear of falling (12–14). The 3-week endpoint was selected as an early feasibility, safety, and adherence checkpoint.

## 3 Case presentations

### 3.1 Patient A

Patient A was a 58-year-old man who began experiencing intermittent palpitations and chest tightness in May 2024, with some episodes accompanied by dizziness and a presyncope sensation. He had four syncopal episodes over approximately one year. Episodes lasted only a few seconds and were followed by rapid recovery without convulsions, urinary incontinence, tongue biting, or prolonged post-event confusion. The most recent episode was associated with fall-related injury. Family video recordings supported transient loss of consciousness and helped exclude seizure-like events.

Cardiac monitoring revealed frequent premature atrial contractions, paroxysmal atrial flutter, and atrial fibrillation; however, Holter monitoring showed a low atrial arrhythmia burden and no documented bradycardia or long pauses. Echocardiography demonstrated preserved left ventricular systolic function, and orthostatic blood pressure testing did not show orthostatic hypotension. Several events were associated with postprandial status, walking, or urination, suggesting autonomic activation and situational triggers. The patient had irregular medical follow-up, limited self-monitoring, and insufficient ability to recognize prodromal symptoms.

### 3.2 Patient B

Patient B was a 74-year-old woman with recurrent dizziness, syncope-like episodes, and transient disturbance of consciousness since September 2024. The initial episode occurred while shopping and was accompanied by dizziness, diaphoresis, blurred vision, and loss of consciousness after sitting down to rest. Later episodes were sometimes associated with vertigo, nausea, limb weakness, and transient loss of consciousness lasting up to approximately 20 min.

Brain imaging showed ischemic cerebrovascular changes. Electrocardiography showed pathological Q waves and ST-segment abnormalities in leads II, III, and aVF. Ambulatory blood pressure monitoring showed blood pressure variability, masked hypertension, and abnormal circadian rhythm. Holter monitoring detected occasional premature atrial and ventricular contractions but no sustained arrhythmia temporally linked to the episodes. Her presentation suggested vasodepressor syncope with multifactorial contributors, including autonomic dysfunction, blood pressure variability, possible cardiac factors, and cerebrovascular comorbidity. She had limited ability to identify prodromes, manage triggers, and use self-protection strategies (Tables 1, 2).

**Table 1:** Timeline of diagnosis, personalized nursing care, and 3-week outcomes.

Time point	Patient A	Patient B
Pre-diagnosis	Recurrent brief syncope over approximately one year; fall-related injury; palpitations and situational triggers.	Recurrent dizziness/syncope-like episodes since September 2024; longer events, BP variability, cerebrovascular changes.
Diagnostic assessment	Head-up tilt-table test with nitroglycerin induced syncope symptoms, BP decrease from 128/83 to 79/39 mmHg, no significant heart-rate change.	Head-up tilt-table test with nitroglycerin induced dizziness, diaphoresis and visual symptoms, BP decreased from 106/75 to 77/44 and then 63/55 mmHg, no significant heart-rate change.
Personalized care planning	Focus on fall prevention, trigger recognition, physical counterpressure manoeuvres, self-monitoring, family engagement.	Focus on prodrome recognition, BP monitoring, anxiety management, graded activity, safety strategies considering age and comorbidity.
0–1 week after discharge	Telephone follow-up; reinforcement of hydration/salt intake, trigger avoidance, and manoeuvre practice.	Telephone follow-up; review of BP log, symptoms, medication-related hypotension risk, and family support.
2–3 weeks after discharge	Telephone plus outpatient review; reinforce daily training and review adherence.	Telephone plus outpatient review; adjust activity plan and psychological support.
3-week outcome	No syncope or fall; improved fear of falling and daily activities.	No syncope or fall; improved anxiety and functional confidence, with residual mild-moderate limitations.

**Table 2:** Diagnostic assessment and head-up tilt-table test findings.

Variable	Patient A	Patient B
Baseline BP before nitroglycerin	128/83 mmHg	106/75 mmHg
Lowest recorded BP during test	79/39 mmHg	77/44 mmHg; subsequent reading 63/55 mmHg
Dominant hemodynamic pattern	Marked hypotension without significant heart-rate change	Marked hypotension without significant heart-rate change
Clinical response	Syncope symptoms and diaphoresis; test terminated	Dizziness, diaphoresis, dimmed/blurred vision; supine position and intravenous fluids relieved symptoms
Interpretation	Tilt-test-confirmed vasodepressor syncope	Tilt-test-confirmed vasodepressor syncope with multifactorial clinical context

### 3.3 Timeline

## 4 Diagnostic assessment

Both patients underwent a standardized syncope work-up including history taking, physical examination, resting ECG, ambulatory rhythm and blood pressure monitoring, echocardiography when indicated, differential diagnosis, and head-up tilt-table testing. In both cases, nitroglycerin-provoked tilt testing induced hypotensive symptoms without an accompanying heart-rate reduction sufficient to suggest a cardioinhibitory response, supporting a vasodepressor phenotype.

## 5 Therapeutic intervention: personalized nursing care pathway

“Nursing interventions” refers to the specific actions delivered within the pathway. The pathway was individualized by linking each patient's risk profile and preferences to evidence-based nursing actions (Table 3).

**Table 3:** Personalized nursing care pathway and individualized intervention priorities.

Domain	Evidence-informed intervention	Individualization for Patient A	Individualization for Patient B
Lifestyle and volume support	Education on hydration, salt intake when not contraindicated, meal regularity, avoidance of heat and dehydration (2, 6).	Emphasis on postprandial and urination-related triggers; reminder card and family-supported hydration schedule.	Emphasis on BP variability and older age; hydration advice combined with BP monitoring and medication review alerts.
Trigger and prodrome management	Identify triggers and initiate protective posture or physical counterpressure manoeuvres at prodrome onset (2, 8).	Training focused on early response to diaphoresis, palpitations, and dizziness; avoid driving when prodromes or recent symptoms occur.	Training focused on blurred vision, dizziness, sweating, and weakness; family instructed to assist with safe sitting/lying.
Physical counterpressure manoeuvres	Leg crossing, handgrip, and lower limb tensing; practice until correct technique is demonstrated (8).	Greater emphasis on fall prevention and confidence restoration because of prior injury.	More gradual training intensity due to age, dizziness, and cerebrovascular/cardiovascular comorbidity.
Orthostatic/tilt training	Home orthostatic training may improve orthostatic tolerance, but evidence is mixed and should be applied cautiously (7, 9).	Daily short sessions increased as tolerated, with symptom stop rules.	Lower initial intensity, family presence during practice, and BP review before progression.
Self-monitoring	Daily BP/heart-rate log and symptom diary; review during follow-up.	Focus on linking diary entries to situational triggers and warning symptoms.	Focus on BP variability, postural symptoms, medication timing, and residual neurological/cardiac warning signs.
Psychological and family support	Assess anxiety and fear of falling; provide reassurance, graded activity, relaxation strategies, and referral if severe (13–15).	Fear of falling prioritized; graded return to routine activity and family supervision.	Anxiety and uncertainty prioritized; structured explanation, reassurance, and family-supported monitoring.

## 6 Follow-up and outcomes

Follow-up was performed for 3 weeks after discharge using telephone contact and outpatient review. Early follow-up was used as a safety and adherence checkpoint because recurrent symptoms, falls, and poor implementation of non-pharmacological measures may occur soon after discharge, and structured syncope follow-up has been associated with improved post-discharge outcomes (10, 16).

During the 3-week follow-up, neither patient experienced recurrent syncope, fall events, emergency visits, or other major discomfort. Both patients reported improved confidence in recognizing prodromes and performing protective responses. Patient A showed greater improvement in fear of falling, consistent with the fall-related injury history. Patient B showed greater improvement in anxiety, although residual limitations persisted because of age, comorbidity, and multifactorial clinical risk.

Lower GAD-7 and FES-I scores indicate improvement. Patient A had a larger reduction in FES-I, consistent with a clinical priority of fall-related fear. Patient B had a higher baseline GAD-7 and a larger absolute reduction in anxiety, consistent with the uncertainty and complexity of her recurrent events (Tables 4, 5).

**Table 4:** EQ-5D-5L health-state and quality-of-life outcomes at baseline and 3 weeks.

Patient	MO pre	SC pre	UA pre	PD pre	AD pre	Index pre	EQ VAS pre	MO 3w	SC 3w	UA 3w	PD 3w	AD 3w	Index 3w	EQ VAS 3w	Delta Index	Delta VAS
A	2	1	3	2	2	0.720	58	1	1	2	1	2	0.906	75	+0.186	+17
B	2	1	4	2	4	0.467	48	2	1	3	2	3	0.651	66	+0.184	+18

MO, mobility; SC, self-care; UA, usual activities; PD, pain/discomfort; AD, anxiety/depression; VAS, visual analogue scale. Higher EQ-5D-5L index and EQ VAS indicate better quality of life.

**Table 5:** Anxiety, fear of falling, and short-term clinical outcomes at baseline and 3 weeks.

Patient	GAD-7 pre	GAD-7 3w	Delta GAD-7	FES-I pre	FES-I 3w	Delta FES-I	Syncope recurrence within 3w	Fall events within 3w
A	8	5	-3	48	32	−16	0	0
B	15	10	−5	44	36	−8	0	0

Lower GAD-7 and FES-I scores indicate improvement. Patient A had a larger reduction in FES-I, consistent with a clinical priority of fall-related fear. Patient B had a higher baseline GAD-7 and a larger absolute reduction in anxiety, consistent with the uncertainty and complexity of her recurrent events.

## 7 Discussion

### 7.1 Main lessons from these cases

These two cases illustrate that vasodepressor syncope nursing care should not be limited to general discharge education. Effective implementation requires translation of guideline recommendations into individualized self-management behaviors. Patient A required emphasis on fall prevention, situational trigger recognition, and confidence restoration. Patient B required closer attention to anxiety, blood pressure variability, comorbidities, and cautious progression of activity. The same evidence base therefore produced different nursing priorities.

### 7.2 Positioning within the current literature

The established evidence base supports education, volume expansion when appropriate, physical counterpressure manoeuvres, and selective orthostatic training (2, 6–9). Outpatient syncope services may also improve outcomes after syncope admission (10, 16). What remains less well described is the nursing process that connects evidence to individualized home implementation, particularly the use of structured follow-up tools, family participation, psychological screening, and patient-reported outcome monitoring. This report addresses that practical gap by describing how individualized goals and measurable outcomes were embedded into a short-term continuity care pathway.

### 7.3 Interpretation of the 3-week endpoint

Short-term absence of recurrence was clinically reassuring in these two cases because both patients had recurrent episodes before admission and Patient A had a fall-related injury. It suggested that the discharge-to-home plan was immediately feasible and that no early fall event, emergency visit, or intolerance of the non-pharmacological programme occurred. However, recurrence in vasovagal syncope is usually evaluated over months or years rather than weeks. In the PC-Trial, recurrent syncope occurred during a mean 14-month follow-up in 50.9% of patients receiving conventional treatment and 31.6% of patients receiving conventional treatment plus physical counterpressure manoeuvre training (8). Earlier post-tilt data also showed that recurrence risk is influenced by the number of previous syncopal episodes (17), and a study of patients with positive head-up tilt testing reported recurrence or typical presyncope in 25.1% during 6–32 months of follow-up, with higher recurrence risk in older patients (18).

Evidence specific to the vasodepressor phenotype remains limited. A recent study of neurally mediated reflex syncope after tilt testing reported that response type was related to both recurrence pattern and quality of life: recurrence was more frequent in the cardioinhibitory group from the second to fourth month, whereas patients with a vasodepressor response had poorer quality-of-life scores (19). These data suggest that a 3-week recurrence-free interval should be interpreted primarily as an early safety, adherence, and self-management signal rather than as evidence of durable recurrence prevention. In the present report, its practical value was that it allowed early verification of prodrome recognition, counterpressure manoeuvre technique, orthostatic/tilt training adherence, blood pressure and heart-rate monitoring, family support, and residual psychological concerns.

### 7.4 Interpretation of quality-of-life, anxiety, and fear-of-falling outcomes

The improvements in EQ-5D-5L, EQ VAS, GAD-7, and FES-I are clinically plausible but should not be interpreted as causal effects. Recurrent vasovagal syncope has been associated with poorer health-related quality of life and higher anxiety and depression compared with non-fainting controls, and psychological distress has been inversely correlated with health-related quality of life (20). Quality of life can also improve after patients are evaluated in a syncope-focused clinical context, even when recurrent fainting occurs during follow-up or when patients are randomized to placebo, suggesting that diagnostic clarification, expert interaction, education, and patient adaptation may themselves contribute to perceived improvement (21).

These mechanisms are consistent with the individualized nursing pathway used here. Education and reassurance may reduce uncertainty about the benign reflex mechanism; trigger identification and action plans may increase perceived control; counterpressure manoeuvre training and safe-positioning instructions may improve self-efficacy; and family participation may increase confidence during daily activities. Patient A's larger FES-I reduction is consistent with his fall-related injury history and the emphasis on fall prevention. Patient B's higher baseline GAD-7 and residual anxiety are consistent with advanced age, comorbidities, blood pressure variability, and uncertainty about multifactorial symptoms. In older adults, syncope has been associated with poorer quality of life, and fear of falling may partly mediate this relationship (22). Therefore, the patient-reported improvements are best interpreted as short-term changes associated with education, structured follow-up, and self-management support; longer follow-up using syncope-specific quality-of-life instruments would be needed to clarify persistence and mechanism (19, 21).

### 7.5 Limitations

This report has several limitations. First, it includes only two cases, without a control group or statistical comparison. Second, the follow-up period was short, so long-term recurrence and durability of self-management behaviours remain unknown. Third, Patient B had multifactorial clinical features, and vasodepressor syncope may not fully explain all symptoms. Fourth, patient-reported outcomes may have been influenced by reassurance, regression to the mean, increased clinical attention, short-term adaptation, or measurement variability. Future prospective studies with larger samples, longer follow-up, and standardized outcome collection are needed.

## 8 Conclusion

An evidence-based personalized nursing care pathway may help patients with vasodepressor syncope translate guideline recommendations into daily self-management behaviours. In these two cases, 3-week follow-up showed no syncope recurrence or falls and suggested improvements in quality of life, anxiety, and fear of falling. In view of recurrence patterns reported over longer follow-up periods, the recurrence-free interval should be regarded as an early safety and adherence observation. Prospective studies with larger samples and longer follow-up are needed to determine whether this pathway reduces recurrence and sustains patient-reported benefits.

## Data Availability

The raw data supporting the conclusions of this article will be made available by the authors, without undue reservation.
